# Melatonin effects on luteinizing hormone in postmenopausal women: a pilot clinical trial NCT00288262

**DOI:** 10.1186/1472-6874-6-8

**Published:** 2006-05-16

**Authors:** Daniel F Kripke, Lawrence E Kline, Farhad F Shadan, Arthur Dawson, J Steven Poceta, Jeffrey A Elliott

**Affiliations:** 1 Scripps Clinic Sleep Center, 10666 North Torrey Pines Road, La Jolla, California 92037, USA; 2 Department of Molecular and Experimental Medicine, The Scripps Research Institute, La Jolla, California 92037, USA; 3 Department of Psychiatry, University of California, San Diego, La Jolla, California 92093-0667, USA

## Abstract

**Background:**

In many mammals, the duration of the nocturnal melatonin elevation regulates seasonal changes in reproductive hormones such as luteinizing hormone (LH). Melatonin's effects on human reproductive endocrinology are uncertain. It is thought that the same hypothalamic pulse generator may both trigger the pulsatile release of GnRH and LH and also cause hot flashes. Thus, if melatonin suppressed this pulse generator in postmenopausal women, it might moderate hot flashes. This clinical trial tested the hypothesis that melatonin could suppress LH and relieve hot flashes.

**Methods:**

Twenty postmenopausal women troubled by hot flashes underwent one week of baseline observation followed by 4 weeks of a randomized controlled trial of melatonin or matched placebo. The three randomized treatments were melatonin 0.5 mg 2.5–3 hours before bedtime, melatonin 0.5 mg upon morning awakening, or placebo capsules. Twelve of the women were admitted to the GCRC at baseline and at the end of randomized treatment for 24-hour sampling of blood for LH. Morning urine samples were collected twice weekly to measure LH excretion. Subjective responses measured throughout baseline and treatment included sleep and hot flash logs, the CESD and QIDS depression self-ratings, and the SAFTEE physical symptom inventory.

**Results:**

Urinary LH tended to increase from baseline to the end of treatment. Contrasts among the 3 randomized groups were statistically marginal, but there was relative suppression combining the groups given melatonin as contrasted to the placebo group (p < 0.01 one-tailed, Mann-Whitney U = 14.) Similar but not significant results were seen in blood LH. There were no significant contrasts among groups in hot flashes, sleep, depression, or side-effect measures and no significant adverse effects of any sort.

**Conclusion:**

The data are consistent with the hypothesis that melatonin suppresses LH in postmenopausal women. An effect related to the duration of nocturnal melatonin elevation is suggested. Effects of melatonin on reproductive endocrinology should be studied further in younger women and in men. Larger studies of melatonin effects on postmenopausal symptoms would be worthwhile.

## Background

In vertebrates, melatonin serves as a hormonal signal of night, and consequently, as a signal of the night's duration through the changing seasons. In a number of mammalian species, the duration of the nocturnal melatonin elevation regulates seasonal changes in reproductive hormones such as luteinizing hormone (LH), follicle-stimulating hormone (FSH), and prolactin [1-4].

Whether melatonin regulates reproductive endocrinology in humans has been uncertain. On the one hand, melatonin was tested with some success as a contraceptive [5]. In a controlled trial, 3 mg. melatonin at bedtime evidently suppressed LH in younger women as compared to placebo, but not among women of menopausal age [6]. Several conditions of inadequate gonadal function or amenorrhea may be associated with enhanced endogenous melatonin production, but not all reports have been consistent [1,7-11]. A prolactin-stimulating effect was noted in women given melatonin 2 mg at 16:00 and 20:00, but there was no significant effect on LH or TSH [12]. Melatonin decreased LH in men in one study, [13] and melatonin impaired sperm production in two young men [14].

Wehr and colleagues subjected experimental subjects to 8 and 14 hours of complete darkness, without attempting to regulate light levels when subjects were out of bed. Dramatic effects of long nights on melatonin, cortisol, and prolactin were demonstrated [15]. The duration of melatonin secretion was prolonged [16]. The duration of the nocturnal prolactin elevation increased, but it was evident from the mean plots that the peak values were higher during short nights, and therefore, it is unclear what the effect of long nights were on total daily prolactin secretion [17]. There were no significant changes in LH from baseline constant routine measurement to measurement after 4 weeks of 14-hour nights; however, since only 3 blood samples were obtained on each occasion, and LH is both highly pulsatile and rather circadian, the sampling did not conclusively address the possibility of effects on LH [17]. Moreover, a man and a woman subjected to 14-hour nights for 15 weeks experienced a disturbing loss of pituitary volume. In that one man, LH and FSH increased during a period of normal nights that followed 15 weeks of long nights, suggesting that secretion of these hormones had been suppressed during the long nights [17].

On the other hand, some controlled trials of melatonin have observed no suppressive effects on reproductive hormones [18]. In a placebo-controlled crossover trial for one month (spring) and 3 weeks (fall), 2 mg melatonin given at 17:00 had no effect on LH [19]. Melatonin 1.5 mg administered at 1600 for 8 days had no effect [20]. Melatonin 10 mg administered at 10 PM had no effect on LH or FSH over 28–40 days [21]. Morning acute administration of 3 mg melatonin was found to amplify LH pulses and area under the curve almost 50% among women in the early follicular phase [22].

In menopausal women, hot flashes are often synchronous with pulsatile release of LH [23,24]. Although hot flashes may not be directly caused by either GnRH or LH, the same hypothalamic pulse generator may initiate both the hot flash and the pulsatile endocrine release. Thus, if melatonin suppressed this pulse generator in menopausal women, it might moderate hot flashes. In our group, Suhner et al. reasoned that by suppressing hot flashes, melatonin might be an effective treatment for menopausal sleep disturbances [25]. In four subjects, they found that 3 mg melatonin at bedtime significantly decreased nocturnal LH secretion after 2 weeks. However, there was no significant reduction in hot flashes. Likewise, in another study of melatonin effects on LH, there was no significant reduction of hot flashes [6].

The current study was planned as an expanded test of the hypothesis that melatonin could suppress LH and relieve hot flashes by suppression of the hypothalamic pulse generator. Wishing to mimic the broader nocturnal melatonin elevation occurring naturally in winter, we gave melatonin not at bedtime but either 2.5–3.0 hours before bedtime or at the time of awakening. We intended to compare effects of evening and morning melatonin administration, which might have opposite effects on circadian timing. Lewy et al. had suggested that excessive melatonin doses might be ineffective stimuli of the circadian system, because blood levels might be elevated for too long [26]. Therefore, the relatively low oral dose of 0.5 mg was selected, considering that even this dose may produce a peak blood concentration well above the physiologic peak [26].

## Methods

Postmenopausal women troubled by hot flashes were recruited for the research by newspaper advertising. Out of 34 women interviewed for the study, 20 signed written informed consent and completed the research. Almost all of the others withdrew before or shortly after signing consent, mostly because their symptoms were not suitable, because they did not wish to adhere to the protocol, or because inspection of arm veins suggested that establishment of an intravenous sampling line might be difficult. A steady dosage of hormone replacement therapy was not considered an exclusion if it did not eliminate hot flashes. The study adhered to the principles of the Helsinki Declaration and was approved and supervised by the local Institutional Review Boards.

**Figure 1:**
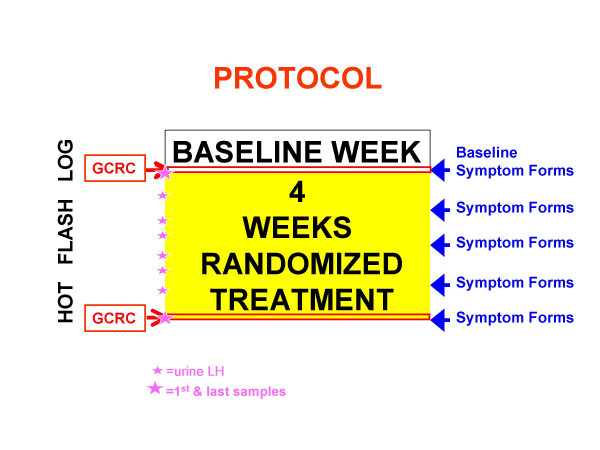
**The experimental protocol**. The protocol consisted of 1 baseline week followed by 4 weeks of randomized treatment. GCRC admissions for blood sampling were scheduled at the end of baseline and at the end of treatment. Symptom forms were obtained weekly and morning urine samples twice weekly. Hot Flashes and Sleep logs were completed daily.

The protocol consisted of one baseline observation week followed by 4 weeks of double-blind randomized treatment with either melatonin 0.5 mg or placebo in identical-appearing capsules (Figure 1). Participants were instructed to take one coded capsule 2.5–3.0 hours before bedtime and one capsule in the morning upon awakening every day for four weeks. Participants received 1) placebo in the evening with 0.5 mg melatonin in the morning, 2) melatonin 0.5 mg in the evening with placebo in the morning, or 3) placebo both evening and morning. The melatonin was Microtonin™ 500 μg manufactured by Cardiovascular Research, Ltd., Concord, CA. The melatonin content of the preparation was informally verified by diluting capsules in water and assaying with the Bühlmann Melatonin Direct RIA. Random assignments were generated by the Consulting Statistician for the Research Pharmacist, who provided the medications in coded bottles for evening and morning administration. Participants, investigators, and General Clinical Research Center staff were blind to treatment assignment.

During the baseline week and throughout the 4 weeks of randomized treatment, participants completed a Daily Log of Sleep and Hot Flashes. This consisted of daily notations of a) bedtime, b) wake-up time, c) the self-estimated percentage of time-in-bed asleep, d) number of hot flashes in 24 hours, and e) a 10 cm. visual analog estimate of daily hot flash severity ranging from "mildest" to "most severe." These self-observations were averaged on a weekly basis. In addition, at the end of each of the 5 weeks, the participants completed two scales for self-rating depression for the previous week, the Center for Epidemiological Studies Depression Scale (CESD) and the Quick Inventory of Depressive Symptomatology self-rating version (QIDS-SR), as well as the SAFTEE, a 96-item symptom inventory designed to monitor adverse effects in clinical trials [27].

At the end of the baseline week, participants were admitted to the General Clinical Research Center for 25 hours, so that venous blood could be sampled repeatedly. An intravenous catheter in a forearm, with long extensions, was established. So far as possible, 2.5 cc blood samples were drawn every 20 min. for 24 hours for assays of luteinizing hormone. Also at this first admission, the baseline CESD, QIDS-SR, and SAFTEE scales were collected, the participants were instructed in urine collection procedures, and the randomized coded medications were provided for 4 weeks of treatment at home. During the randomized treatment, participants collected a first morning urine specimen twice a week, measuring the volume, recording the collection intervals, and freezing aliquots, so that urinary LH excretion could be examined. On the last day of the 4 weeks of randomized treatment, participants were readmitted to the General Clinical Research Center for repeated 24-hour blood sampling and collection of all data forms. Participants who completed the study were paid $440, or pro-rata for early discontinuation.

The staff experienced considerable difficulty in maintaining the venous catheters and obtaining blood specimens. For the first 12 participants, of 1898 planned samples, only 1090 samples were successfully frozen. However, at least a few samples were obtained from each of the 12 participants, making it possible to estimate daily mean LH blood concentration. Because of the missed samples and the small number of subjects completing the blood sampling procedures, it was not possible to analyze the parameters of pulsatile LH secretion.

We had originally planned to complete study of 30 subjects to have reasonable power for the endocrine studies, recognizing that for the sleep, hot flashes, and symptom data, a much larger number of subjects would be desirable for adequate power [28]. After 13 participants had completed the protocol (blood LH from one could not be used), it was unfortunately necessary to discontinue the inpatient studies, because of loss of GCRC funding. An additional 7 subjects completed the protocol entirely at home, collecting urine but no blood samples. Resources did not permit recruitment of the final 10 subjects planned.

### Assays

LH levels in blood and urine were measured using DSL-10-460 Active^R ^LH Elisa kits (Diagnostic System Laboratories, Inc, Webster, TX.) This is an enzymatically amplified "one-step" sandwich-type immunoassay. The manufacturer states that there is no detectable cross-reactivity with TSH, FSH, and hCG. Blood samples were measured by direct addition of 50 μL serum, whereas urine samples were measured by diluting 1:1 with zero standards (each at 25 μL). For accuracy, above-scale LH urine samples (> 180 mIU/ml) were re-assayed at increased dilutions. The sensitivity of the EIA is 0.1 mIU/ml. At mean LH concentrations of 3.2 mIU/ml and 30.8 mIU/ml, intra-assay coefficients of variation were 2.2% and 4.9% respectively, and inter-assay CVs were 2.9% and 4.8% respectively. Urinary rates of LH excretion (mIU/hr) were computed by multiplying the urine LH concentration (mIU/ml) by the urine excretion rate (ml/hr) calculated over the interval between voidings. Measured urinary LH excretion was quite erratic from day to day, with the daily standard deviation being 69% of the mean. Therefore, for each subject, daily urine LH mIU/hr values outside a range of ± 2SD were ignored, in most cases leaving 8–9 values for computation of slopes. The first and last values meeting the ± 2SD criteria were also selected to explore changes in urinary LH excretion during treatment.

### Statistics

Contrasts between groups were examined using ANOVA, with the initial score as the covariate. When appropriate due to the distributions, these results were confirmed with nonparametric analyses, which in the case of LH measures, were applied to change scores.

## Results

The 20 participants completing the study had a mean age of 59 years (range 51–69).

### Luteinizing hormone

**Figure 2:**
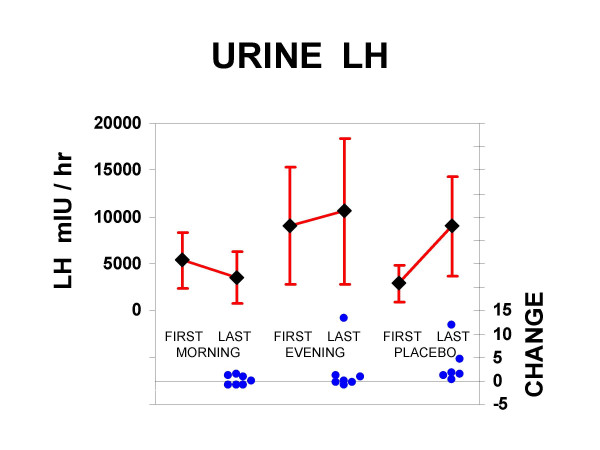
**Urinary LH results**. Above, the means and 95% confidence intervals of urinary luteinizing hormone excretion are shown in units of mIU/hr. For the morning melatonin, evening melatonin, and placebo groups, both the initial and final samples are illustrated, with a line representing the direction of change. Below, the change ratios from first to last sample are plotted as filled circles for each subject, ranging from a 90% decrease to a 13-fold increase.

As expected due to random group assignment, baseline values of urinary LH excretion among the 3 groups were not significantly different, but the group which would be randomized to receive melatonin in the evening averaged substantially higher excretion than the other two groups. Therefore, the percent changes between first and last samples divided by the initial level of the first sample were examined to determine effects of randomized treatments. Contrasting urinary LH excretion among the 3 groups, these change scores adjusted for the initial level were marginally different (Kruskal-Wallace p < 0.06, DF = 2); however, combining the two melatonin groups because of the small N and the prospective prediction that melatonin at both times would suppress LH, there was suppression among those given melatonin as contrasted to the placebo group (p < 0.01 one-tailed, Mann-Whitney U = 14.) Likewise, the slopes of the urine LH excretion (including up to 8 bi-weekly samples) were lower among those receiving melatonin than placebo (p < 0.06, one-tailed, Mann-Whitney U = 22). There were no significant differences between morning and evening melatonin results. Urinary luteinizing hormone results are shown in Figure 2.

**Figure 3:**
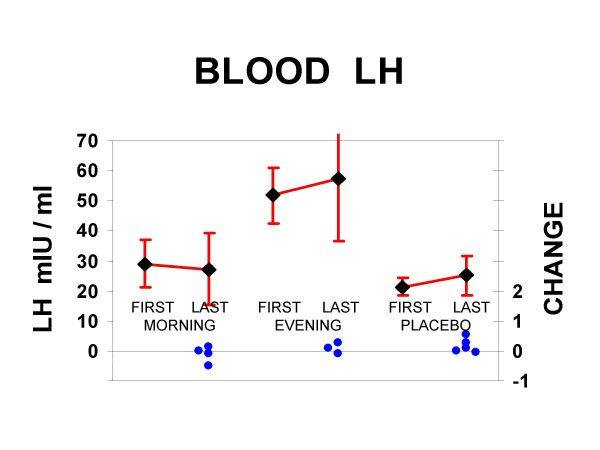
**Blood LH results**. Above, the means and 95% confidence intervals of blood luteinizing hormone are shown in mIU/ml. For the morning melatonin, evening melatonin, and placebo groups, both the initial and final samples are illustrated, with a line representing the direction of change. Below, the changes from first to last sample are plotted as filled circles for each subject, ranging from 54% decrease to a 49% increase. Note that although the dispersion of change scores was much smaller for blood than for urinary measures of LH, the small sample sizes for blood LH provided weaker power to contrast groups.

In the smaller subgroups which provided blood LH samples, the averaged baseline concentrations were 55 mIU/ml among those that would randomly be given melatonin in the evening, 28 mIU/ml among those to receive morning melatonin, and 23 mIU/ml among those receiving only placebo (Kruskal-Wallace p = 0.02, DF = 2). For blood LH, change scores were not significantly different among the 3 groups, or when contrasting the two melatonin groups combined with the placebo group. On overall average, mean blood LH increased slightly (not significantly) from the first to the last collection, with a 17% ± SD 27% increase in the placebo group, a 9% ± SD 19% increase in the evening melatonin group, and an 11% ± SD 24% decrease in the morning melatonin group. For the observed differences between the morning and evening melatonin groups and the placebo group in blood LH change scores, 14 cases in each group would be needed for 82% power to distinguish the groups, modeling ANOVA. Using the observed urine data, 27 cases in each group would be needed for 80% power. Blood LH results are shown in Figure 3.

The rank-order correlation of average blood LH at baseline versus the first urine excretion sample was r_S _= 0.84 (P < 0.001). The final treatment measures were correlated r_S _= 0.73 (P < 0.01). The adjusted changes in urine LH excretion and blood LH concentration were positively but not significantly correlated.

### Hot flashes

Reported hot flashes per day averaged 8.9 in the baseline week and 7.8 in the final week of randomized treatment. There was no significant difference between groups in hot flashes change, but the trend was for the placebo group to report the most improvement (contrary to prediction). Similarly, reported severity on the visual-analog scales dropped from 47 at baseline to 37 in the final week, with the greatest drop in the placebo group, though group contrasts were not significant. Likewise, changes were not significant between groups in the product of the reported number of hot flashes times the reported severity. Changes in hot flashes from baseline to the end of treatment were not significantly correlated with changes in blood or urinary LH over the same interval.

### Sleep

In the baseline week, the participants reported an average bedtime of 10:52 PM and an average uptime of 06:41 AM, with an average of 85% of the time-in-bed spent asleep. Though reported sleep percent increased to 88% at the end of treatment, there were no significant contrasts between treatment groups. The prediction that evening melatonin administration would phase-shift sleep times earlier than would morning melatonin (based on the melatonin phase-response curve) [29] was not confirmed.

### Mood

On the CESD, mood improved from an average score of 7.5 at baseline to 6.9 in the final week. A CESD score >16, which is a sensitive screening criterion for major depression, was reported by 4 women at baseline, but only by 2 at the end of treatment. Perhaps because the group which would receive morning melatonin included the participant with the highest CESD (which improved during the study), that group showed the greatest improvement, but the contrasts between groups were not significant. Similarly, the average QIDS scores improved from 5.2 to 4.3 from baseline to the end of randomized treatments, with no significant differences between groups.

### Adverse effects

There were no clinically significant or severe adverse effects reported during randomized treatment with melatonin or placebo. Some women felt that the medication caused mild sleepiness, but it was tolerable. The SAFTEE symptom inventory displayed a 9.2% average reduction of diverse medical symptom reports from baseline to the end of treatment. Seventeen classes of symptoms and 10 individual items related to sleepiness and mood were examined, but there were no significant contrasts between randomized groups.

## Discussion

To summarize, this trial tended to confirm a preliminary study of Suhner et al. [25] and support the hypothesis that melatonin given to postmenopausal women can suppress luteinizing hormone as compared to placebo. This result was statistically marginal. The amount of suppression compared to placebo was very modest, considering the high levels of secretion among women of this age. Indeed, since participants receiving placebo showed higher LH at the end of treatment than at baseline, suppression in the group receiving melatonin in the evening was suggested merely by a smaller percentage increase than in the placebo group. The directions of change were similar in both blood and urine LH. Urine results were far more erratic, but perhaps because data for urine LH were available for more subjects, the contrasts were somewhat more statistically significant. Because fewer subjects than planned were studied and the statistical outcome was not conclusive, these results certainly need replication.

It was interesting to observe that morning melatonin was associated with more suppression of LH than evening melatonin, though not significantly so. Perhaps melatonin administration 4–5 hours before bedtime might have served to make the evening onset of melatonin more dramatically early and more effective. The investigators had not chosen to give evening melatonin 4–5 hours before bedtime, because it was believed that evening sedation and a possibly unwanted advance in sleep timing might be more severe with earlier administration.

It would be important to examine melatonin effects on the reproductive endocrinology of women of other ages and effects on men. Due to the excess LH secretion of postmenopausal women, effects of melatonin were not expected to be harmful to this group. However, if melatonin interferes with the reproductive endocrinology of women attempting to have children or suppresses LH (and thus testosterone) among men, such effects might be quite unwelcome. Since melatonin is becoming popular in the treatment of children with developmental problems, it would also be important to explore whether melatonin interferes with their endocrine development.

The observations were consistent with the hypothesis that photoperiodic mechanisms are active in human physiology. The timing chosen (well before bedtime or after arising) and the low dosage of melatonin, designed to simulate effects of a natural winter photoperiod upon the melatonin profile, may explain why we were able to demonstrate this marginal melatonin effect on LH, when other studies have not had similar results. Both the before-bedtime and morning administration would tend to extend the duration of the nightly melatonin elevation. Possibly, other studies have failed to document an effect of melatonin on LH because bedtime dosing or use of higher doses creates 24-hour melatonin profiles less similar to those produced by a natural winter photoperiod.

Nevertheless, it might be possible to further optimize timing and dosage.

## Conclusion

The results of this study did not support our hypothesis that melatonin would improve sleep and suppress hot flashes among symptomatic postmenopausal women, but there were no significant adverse effects, and LH was possibly suppressed. There were too few subjects to provide adequate power for exploration of symptomatic effects, especially considering the well-known placebo responses in studies of this type [28]. Therefore, larger studies of melatonin effects on postmenopausal women with hot flashes are needed.

## Competing interests

The author(s) declare that they have no competing interests.

## Authors' contributions

Dr. Kripke conceived the study, admitted the participants to the GCRC, and performed data analyses. Drs. Kline, Shadan, Dawson, and Poceta contributed to study planning and administration and provided clinical coverage. Dr. Elliott contributed to experimental design, performed the luteinizing hormone assays, and calculated urinary excretion rates. All authors contributed to manuscript preparation.

## Pre-publication history

The pre-publication history for this paper can be accessed here:


